# Chlorine gas poisoning by trichloroisocyanuric acid and respiratory failure: a case report of a 49-year-old patient

**DOI:** 10.3389/fmed.2025.1676596

**Published:** 2025-12-10

**Authors:** Yamei Wang, Jun Meng, Xiaowei Tian, Chuihua Sun, Lu Liu, Xiaojuan Sun

**Affiliations:** 1 Department of Occupational Diseases, Weifang People’s Hospital, Weifang, Shandong, China; 2 Department of Critical Care Medicine, Weifang People’s Hospital, Weifang, Shandong, China

**Keywords:** trichloroisocyanuric acid, respiratory failure, case report, corticosteroid therapy, management

## Abstract

**Background:**

Trichloroisocyanuric acid (TCCA) is a potent disinfectant and bleaching agent widely used in industrial applications. When TCCA comes into contact with water, chlorine gas and hypochlorous acid are produced. Although acute respiratory distress syndrome (ARDS) induced by chlorine gas inhalation is rare, if untreated, it can progress to severe respiratory failure. Currently, no standardized glucocorticoid dosing protocol exists for ARDS management in such cases.

**Case presentation:**

This report details the case of a 49-year-old male who developed ARDS following chlorine gas inhalation during an occupational TCCA incident. Upon admission, the patient exhibited shortness of breath, coughing, and dyspnea. Initial laboratory tests revealed leukocytosis and elevated inflammatory markers. Imaging showed bilateral patchy ground-glass opacities, and arterial blood gas analysis indicated severe hypoxia. The patient was treated with non-invasive mechanical ventilation and a graded corticosteroid regimen, leading to gradual improvement in his clinical condition. During this course, the patient developed fever, frequent dry cough, and occasional sputum production. Sputum cultures identified Candida albicans as the pathogen, prompting a shift in treatment to include fluconazole for antifungal therapy, cefoperazone-sulbactam for antibacterial coverage, and continued corticosteroid therapy. The patient recovered progressively and was discharged on the 51st day without complications.

**Conclusion:**

Chlorine gas inhalation can result in severe ARDS, underscoring the need for early diagnosis and prompt intervention. This case underscores the importance of a graded corticosteroid regimen in combination with non-invasive mechanical ventilation in ARDS management. Additionally, fluconazole and cefoperazone effectively address secondary pulmonary fungal infections caused by Candida albicans.

## 1 Introduction

Trichloroisocyanuric acid (TCCA) is a potent chlorine-based disinfectant renowned for its strong bactericidal and bleaching properties. It is widely applied in industries such as swimming pool and drinking water sanitation, aquaculture, and consumer chemicals (1–3). In damp or high-temperature environments, TCCA decomposes, generating heat, releasing chlorine gas, and potentially leading to violent explosions (4, 5). The toxicity primarily stems from chlorine poisoning and the damage caused by the chemical products released (6, 7). Chlorine gas, with its highly oxidizing and corrosive properties, can cause acute poisoning and tissue damage, especially to the eyes, skin, and mucous membranes (8–10). The respiratory system is particularly vulnerable to chlorine inhalation, with the severity of lung injury depending on the gas concentration, exposure duration, and ventilation conditions (11, 12).

The toxic effects of TCCA are mainly attributed to the high concentration of chlorine gas released during its decomposition (11). Chlorine reacts with moisture in bronchial cells to form hydrochloric acid and hypochlorous acid, causing bronchospasm and lung damage (13, 14). The core mechanism of chlorine gas toxicity lies in its potent oxidative nature. Chlorine induces oxidative damage to mitochondrial DNA (mtDNA), leading to the excessive generation of reactive oxygen species (ROS) that disrupt mitochondrial function (15, 16). This damage affects the expression of lung DNA glycosylase OGG1 and nitric oxide synthase, further exacerbating mtDNA oxidative damage, altering the mitochondrial proteome, and promoting the formation of mitochondrial permeability transition pores, which facilitate cell death (17, 18). Hypochlorous acid, a byproduct of oxidation, inhibits antioxidant enzymes like catalase and glutathione peroxidase, intensifying cellular damage (19, 20). Injury to alveolar epithelial cells and the endothelial cells of capillaries triggers the release of inflammatory mediators (e.g., TNF-α, IL-1, IL-6, IL-8), which activate neutrophils and macrophages to release additional proteases and ROS such as superoxide, hydrogen peroxide, and hydroxyl radicals (14, 21, 22). This inflammatory cascade damages the alveolar epithelium, disrupts the basement membrane, impedes fluid resorption, and promotes the accumulation of proteins and blood cells in the alveolar spaces. Prolonged inflammation and oxidative stress lead to abnormal lung tissue repair, resulting in fibrotic hyperplasia, which manifests as irreversible restrictive ventilatory dysfunction and impaired gas exchange (16, 17).

Clinically, low-level exposure to chlorine gas may be asymptomatic, while higher concentrations can induce symptoms such as cough, chest tightness, mediastinal oscillation, acute respiratory distress syndrome (ARDS), respiratory failure, and even cardiopulmonary arrest (23, 24). Severe chlorine gas poisoning may also lead to the rare complication of mediastinal emphysema (25). For example, Li et al. reported 27 patients who experienced symptoms from chlorine gas inhalation due to TCCA, with 11 diagnosed with severe poisoning. Of these, two young patients developed mediastinal emphysema (12). A previous study also documented a 26-year-old woman who developed mediastinal emphysema after inhaling chlorine gas from a household cleaning product. Other complications of chlorine gas poisoning include chemical pneumonitis and ARDS (26–28). As no specific antidote exists for inhalation-induced lung injury, management is generally supportive, with targeted therapies such as lung-protective ventilation, prone positioning, nebulized bronchodilators, and systemic corticosteroids playing beneficial roles (29–31).

Corticosteroids, potent anti-inflammatory agents, inhibit the synthesis of proinflammatory mediators and modulate systemic immune responses (30). They are commonly used in the treatment of conditions such as COVID-19, viral pneumonia, community-acquired pneumonia, and ARDS (32–35). Recent clinical guidelines recommend the conditional use of corticosteroids in patients with ARDS, based on moderate evidence (36). A meta-analysis indicated that corticosteroids, including methylprednisolone and dexamethasone, are associated with reduced mortality in patients with ARDS (37). However, no randomized controlled trials have compared the efficacy of different corticosteroid doses in treating lung injury, and initial steroid dosing varies among physicians (38). This study presents a case of chlorine gas poisoning from TCCA exposure. Although chlorine gas inhalation can be fatal, timely intervention can lead to recovery. Given that corticosteroids reduce proinflammatory mediators and prevent diffuse alveolar damage, the patient was treated with high-dose methylprednisolone in a tapering regimen, alongside non-invasive ventilatory support. As the glucocorticoid dosage was gradually reduced, the patient developed new symptoms, including fever and purulent sputum, suggesting a secondary infection. Sputum cultures confirmed Candida albicans infection. Clinicians should remain vigilant for emerging symptoms, such as fever, purulent sputum, or new and worsening radiographic findings.

## 2 Case presentation

**FIGURE 1:**
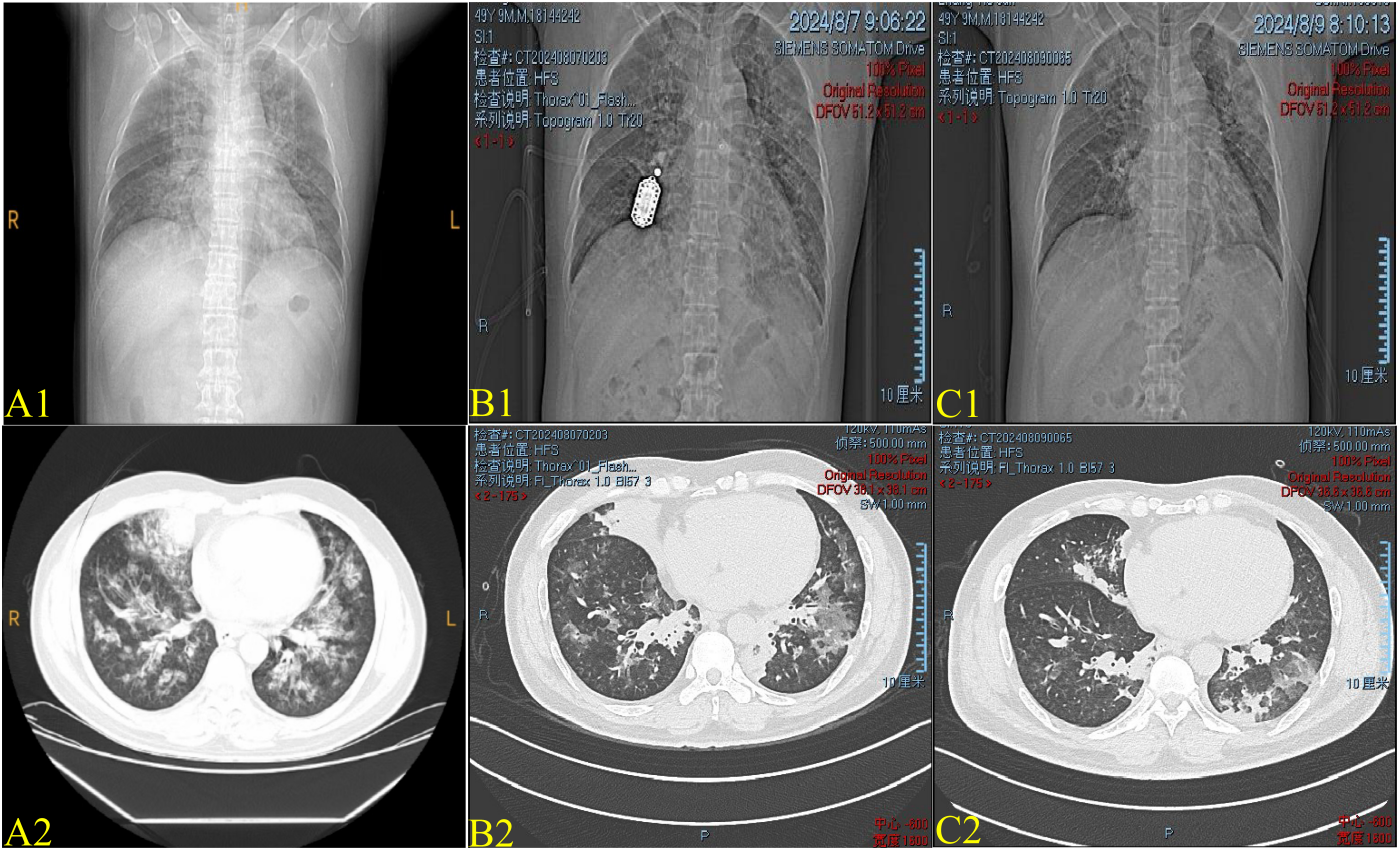
**(A1–C1)** Chest X-ray on Day 1, Day 3, and Day 5. **(A2–C2)** Images of chest computed tomography (CT) on Day 1, Day 3, and Day 5. **(A1,A2)** By Day 1, the Chest X-ray and CT images show diffuse consolidation in both lungs, predominantly in the lower regions, indicative of acute lung injury. **(B1,B2)** By Day 3, the lesions show rapid absorption, suggesting a positive response to treatment. **(C1,C2)** By Day 5, the lesions have almost completely resolved, reflecting significant recovery and resolution of lung injury.

A 49-year-old male, engaged in poultry greenhouse disinfection, was resting in a sealed 10-square-meter room containing TCCA powder. Due to excessively high temperatures, the powder exploded violently, releasing a substantial volume of smoke. The patient was awakened by the explosion’s sound and the pungent odor, prompting him to immediately flee the room. His condition deteriorated rapidly, with symptoms including headache, dizziness, tearing, shortness of breath, coughing, pink frothy sputum, and dyspnea. Two hours later, he was transferred to a local hospital for treatment. Due to breathing difficulties, the patient was unable to lie flat and assumed a sitting position. On day 1, computed tomography (CT) scan revealed interlobular septal thickening and patchy ground-glass opacities in both lungs, indicating acute lung injury (Figures 1A1, A2). The patient was treated with moxifloxacin and cefuroxime for infection, methylprednisolone for inflammation, and alternating eye treatments with deproteinized bovine blood extract gel, levofloxacin eye drops, and bromofenac sodium eye drops. Despite slight improvement in symptoms, the patient remained unable to lie flat. He was subsequently admitted to our department with a diagnosis of chlorine poisoning, chemical pneumonia, and ARDS. Since the onset of symptoms, the patient had exhibited poor mental status, loss of appetite, and sleep disturbances. He had a 3-year history of hypertension but had not been on any antihypertensive medications.

Upon arrival at the emergency department, the patient’s vital signs were as follows: heart rate 90 beats per minute, temperature 37.3 °C, respiratory rate 19 breaths per minute, and blood pressure 138/81 mmHg. Eye examination revealed secretions on both eyelids, conjunctival congestion, and corneal opacity, with no yellow staining of the sclera. A small area of skin damage was noted on the right anterior chest, but no abnormalities were observed in the mucosa. Chest expansion was restricted due to pain, and respiratory auscultation revealed extensive diffuse dry and wet rales in both lungs, with no other notable physical findings. Initial blood tests showed the following: white blood cells (WBC) 24.98 × 10^9^/L, neutrophil granulocytes (GRAN) 23.78 × 10^9^/L, total protein (TP) 56.4 g/L, serum albumin (ALB) 31.1 g/L, C-reactive protein (CRP) 122.0 mg/L, D-dimer 2.97 μg/mL, prothrombin time (PT) 14.9 s, prothrombin activity (PT%) 69.4%, fibrinogen (FIB) 5.12 g/L, activated partial thromboplastin time (APTT) 30.4 s, and thrombin time (TT) 14.1 s. Biochemical results showed AST 47 U/L, ALB 36.0 g/L, glucose (GLU) 8.0 mmol/L, urea (UREA) 8.7 mmol/L, LDL-C 1.83 mmol/L, Apo-A 0.71 g/L, LDH 377 U/L, CK 597 U/L, and CK-MB 8.26 ng/mL. Urinalysis indicated proteinuria (1+), but other tests, including hepatitis B markers, troponin, BNP, and procalcitonin, were unremarkable. After receiving high-flow oxygen via a nasal cannula (6 L/min), arterial blood gas analysis revealed a pH of 7.43, PaCO_2_ of 38.8 mmHg, and PaO_2_ of 71.1 mmHg (Table 1). The PaO_2_/FiO_2_ ratio was 158, confirming severe ARDS. The patient was promptly placed on non-invasive ventilatory support with the following settings: positive end-expiratory pressure (PEEP) 12 cmH_2_O, FiO_2_ 1.0, tidal volume 430 mL, and respiratory rate 15 breaths per minute. The treatment regimen included intravenous moxifloxacin and cefuroxime for infection, glutathione for antioxidant therapy, Ginkgo biloba extract to improve circulation, and nebulized treatments with budesonide, formoterol fumarate, and ambroxol for sputum clearance and asthma relief. For ocular management, the patient received bromfenac sodium eye drops, levofloxacin eye drops, deproteinized bovine blood extract eye drops, and recombinant bovine matrix cell growth factor eye gel to address conjunctival congestion, corneal epithelial defects, and mild edema.

**TABLE 1:** Summary of patient’s laboratory test results.

Variables	Augst 6, 2024 (Day 2)	Augst 7, 2024 (Day 4)	Augst 10, 2024 (Day 6)	Augst 13, 2024 (Day 9)	Augst 16, 2024 (Day 12)	Augst 21, 2024 (Day 17)	Augst 27, 2024 (Day 23)	Augst 28, 2024 (Day 24)	Augst 29, 2024 (Day 25)	September 6, 2024 (Day 33)	September 19, 2024 (Day 46)	Reference rage
White blood cell count (×10^9^/L)	24.98	19.53	13.56	15.66	14.87	11.88	12.48	–	–	11.66	12.56	3.5–9.5
Neutrophile granulocyte (×10^9^/L)	23.78	17.64	10.69	11.73	10.34	7.54	10.53	–	–	7.03	7.78	1.8–6.3
Monocyte (×10^9^/L)	1.8	0.78	0.84	0.81	0.62	0.67	0.27	–	–	0.75	0.57	3–10
Lymphocyte (×10^9^/L)	0.75	1.11	2.03	3.1	3.87	3.61	1.68	–	–	3.86	4.17	1.1–3.2
Hemoglobin (g/L)	153	141	142	149	147	143	158	–	–	150	144	130–175
Platelet (×10^9^L)	262	250	257	295	280	233	282	–	–	279	244	120–350
CRP (mg/L)	122.0	38.1	13.5	2.3	0.7	0.4	2.2	–	–	1	1.6	0–8
Procalcitonin (ng/mL)	–	0.21	–	0.07	–	–	0.25	0.37	–	–	–	0–0.5
ALT (U/L)	27	28	–	48	62	52	42	–	–	40	51	0–50
AST (U/L)	47	30	–	24	22	16	17	–	–	23	24	0–40
Creatine phosphokinase (U/L)	597	163	–	281	82	37	–	171	–	29	24	50–310
Lactate dehydrogenase (U/L)	377	373	–	321	313	233	–	228	–	240	234	120–250
Hypersensitive troponin T (pg/mL)	<1.0	<1.0	–	<1.0	<1.0	1.1	–	1.5	–	–	–	0–19.8
BNP (pg/mL)	25.5	–	–	–	–	–	–	30.9	30.9	30.1	–	0–100
Creatinine (μmol/L)	65	60	–	68	64	58	–	60	–	71	63	51–97
Carbamide (mmol/L)	8.7	10.2	–	9.1	8.7	10	–	9.3	–	12.5	7.6	3.1–8.0
Uric acid (μmol/L)	290	237		170	127	186	–	256	–	303	263	208–428
Amylase (U/L)	68	–	–	–	–	–	–	–	–	69	64	35–135
Arterial blood gas analysis	–	–	–	–	–	–	–	–	–	–	–	–
pH	7.43	–	–	–	–	–	–	7.40	–	–	–	7.35–7.45
pCO_2_ (mmHg)	38.8	–	–	–	–	–	–	43.8	–	–	–	35–45
pO2 (mmHg)	71.1	–	–	–	–	–	–	108	–	–	–	80–100
Lactate (mmol/L)	1.2	–	–	–	–	–	–	2.3	–	–	–	0.6–2.0
Coagulation test	–	–	–	–	–	–	–	–	–	–	–	–
Prothrombin time (s)	14.9	15.0	–	13.7	–	12	–	12.5	–	–	–	10.0–14.0
Prothrombin activity (%)	69.4	69.1	–	82.7	–	99.9	–	94.43	–	–	–	80–130
Plasma fibrinogen (d/L)	5.02	4.09	–	3.05	–	3.36	–	2.75	–	–	–	2.0–4.0
Activated partial thromboplastin time (s)	30.4	27.7	–	27.9	–	27.6	–	31.3	–	–	–	22.0–38.0
Thrombin time (s)	14.1	14.0	–	16.8	–	15.4	–	16.2	–	–	–	14.0–21.0
D-dimer (μg/ml)	2.97	1.52	–	1.03	–	0.52	–	0.46	–	–	–	0.00–1.00
Urine routines	–	–	–	–	–	–	–	–	–	–	–	–
Urine specific gravity	1.026	–	1.027	–	–	–	–	–	–	–	–	–
Urinary protein (g/L)	+	–	−	–	–	–	–	–	–	–	–	–
Ketone body (mmol/L)	+	–	−	–	–	–	–	–	–	–	–	–
Vitamin C (mmol/L)	+++	–	+	–	–	–	–	–	–	–	–	–
Urocholinogen (umol/L)	−	–	−	–	–	–	–	–	–	–	–	–
Bilirubin (umol/L)	−	–	−	–	–	–	–	–	–	–	–	–

WBC, white blood cells; GRAN, neutrophile granulocyte; CRP, C-reactive protein; BNP, brain natriuretic peptide; ALT, aalanine aminotransferase, AST, aspartate aminotransferase; PT, prothrombin time; FIB, fibrinogen; APTT, activated partial thromboplastin time; TT, thrombin time.

**FIGURE 2:**
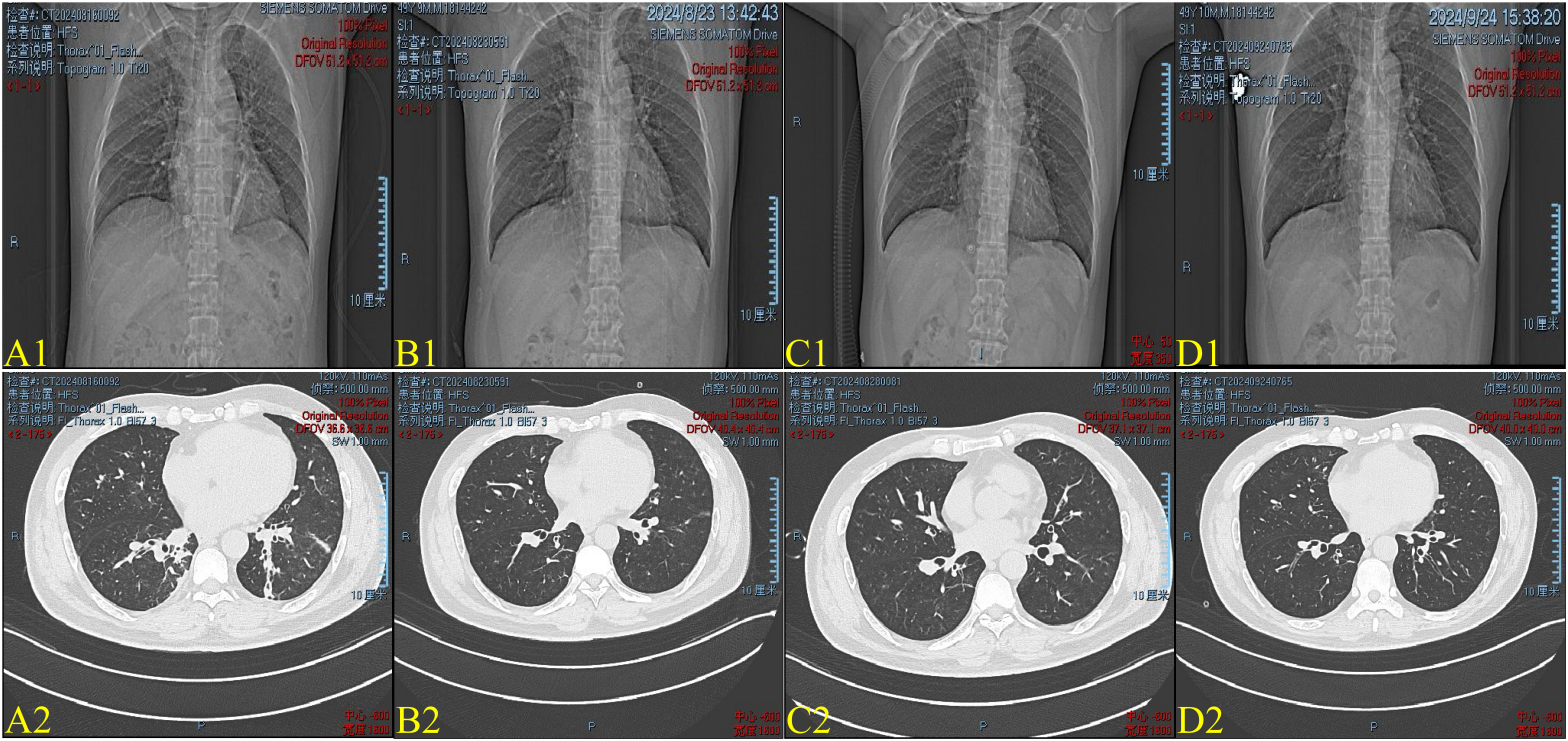
**(A1–D1)** Chest X-ray on Day 12, Day 19, Day 25, and Day 51. **(A2–D2)** Images of chest computed tomography (CT) on Day 12, Day 19, Day 25, and Day 51. **(A1,A2)** On Day 12, the Chest X-ray and CT images show diffuse consolidation in both lungs, primarily in the lower regions. **(B1,B2)** By Day 19, the lesions show rapid absorption, indicating a favorable response to treatment. **(C1,C2)** By Day 25, the lesions are nearly resolved, suggesting substantial recovery and resolution of lung injury. **(D1,D2)** By Day 51, the lesions have almost completely resolved, indicating further recovery and complete resolution of lung injury.

On day 3, a repeat CT scan revealed patchy ground-glass opacities in both lungs (Figures 1B1, B2), with the SpO_2_/FiO_2_ ratio measuring 207. The patient received intravenous methylprednisolone sodium succinate (200 mg daily), cefuroxime (1.5 g every 8 h), and oral coenzyme Q (10 mg three times daily). By the fourth day, re-examination showed WBC 19.53 × 10^9^/L, GRAN 17.64 × 10^9^/L, LDH 373 U/L, CK 597 U/L, CK-MB 8.26 ng/mL, CRP 38.1 mg/L, D-dimer 1.52 μg/mL, PT 15.0 s, PT% 69.01%, FIB 4.09 g/L, APTT 27.7 s, and TT 14.0 s. On day 5, a CT scan revealed reduced ground-glass opacity and minimal pleural effusion (Figures 1C1, C2), with the SpO_2_/FiO_2_ ratio improving from 207 to 229. Given the patient’s improvement, the glucocorticoid dosage was reduced from 200 mg daily to 160 mg daily. By day 6, laboratory results showed WBC 13.56 × 10^9^/L, GRAN 10.69 × 10^9^/L, TP 56.4 g/L, and ALB 31.1 g/L. On day 8, the SpO_2_/FiO_2_ ratio increased to 257, reflecting further stabilization. The glucocorticoid dosage was reduced to 80 mg daily. Follow-up results indicated WBC 15.66 × 10^9^/L, GRAN 11.73 × 10^9^/L, TP 54.1 g/L, and ALB 29.3 g/L. By day 12, a chest CT showed small bilateral pleural effusions and partial improvement in bilateral pneumonia, confirming the treatment’s effectiveness (Figures 2A1, A2). On day 12, laboratory results indicated WBC 14.87 × 10^9^/L, GRAN 10.34 × 10^9^/L, TP 54.1 g/L, and ALB 29.3 g/L, with the SpO_2_/FiO_2_ ratio rising to 334, indicating marked improvement in oxygenation. The methylprednisolone sodium succinate dosage was further reduced to 40 mg daily. Due to decreased albumin levels, a high-protein, nutrient-dense diet was recommended. A consultation with the nutrition department led to the prescription of 40 g of whole protein nutritional supplements and 10 g of whey protein per serving.

Given the patient’s stable condition and improvement in various parameters, including WBC 11.88 × 10^9^/L, GRAN 7.54 × 10^9^/L, and SpO_2_/FiO_2_ ratio of 334 on day 17, the medication regimen was adjusted from intravenous methylprednisolone 40 mg to oral administration of 20 mg. The following day, the patient began experiencing frequent dry cough with occasional small amounts of sputum. A bacterial sputum test identified a fungal infection, confirming a secondary Candida albicans infection. On day 19, a CT scan revealed solid micronodules in both lungs, primarily consisting of proliferative lesions (Figures 2B1, B2). Fluconazole was initiated for antifungal treatment, while corticosteroid therapy was continued, and cefoperazone-sulbactam was added for antibacterial coverage. By day 25, the CT scan showed multiple solid micronodules in both lungs, most of which were hyperplastic (Figures 2C1, C2). No significant abnormalities were observed in arterial blood gas analysis, myocardial enzyme spectrum, or BNP. Due to the patient’s bronchial asthma (reactive airway dysfunction syndrome) following poisoning, treatment with budesonide-formoterol, tiotropium bromide, and acetylcysteine was administered for cough relief, asthma control, and sputum clearance. On day 46, laboratory results showed K 3.97 mmol/L, TP 58.2 g/L, ALB 36.5 g/L, HDL-C 0.69 mmol/L, LDL-C 2.72 mmol/L, LDH 240 U/L, CK 29 U/L, WBC 12.56 × 10^9^/L, LYMF 4.17 × 10^9^/L, GRAN 7.78 × 10^9^/L, RBC 4.60 × 10^12^/L, and CRP 1.6 mg/L (Table 1). Pulmonary function testing revealed extremely severe mixed ventilatory dysfunction with normal diffusion capacity. On day 51, the CT scan showed new localized patellar ground-glass opacities in the middle lobe of the right lung, along with solid micronodules in both lungs, primarily consisting of proliferative foci (Figures 2D1, D2). With active treatment, the patient’s condition continued to improve. The patient was advised to rest adequately, avoid exposure to toxic substances, and continue taking methylprednisolone, coenzyme Q10, and betamethasone sustained-release glycyrrhizin tablets, in addition to inhaling tiotropium bromide and budesonide-formoterol powder.

## 3 Discussion

Trichloroisocyanuric acid (TCCA) is a harmful gas commonly produced in chlorine disinfection environments, with chlorine gas released during its decomposition being the primary cause of inhalation-induced lung injury (4, 5). This gas can irritate the eyes, skin, and respiratory system, resulting in symptoms that range from mild to severe. The severity of lung injury is determined by the concentration of chlorine gas, exposure duration, and the minute ventilation of the exposed individual (11). Low exposure levels (below 15 ppm) typically cause mucosal irritation without symptoms, while higher concentrations (above 30 ppm) may result in chemical pneumonitis and bronchiolitis obliterans. Exposure to concentrations exceeding 400 ppm can cause immediate respiratory arrest (39, 40). Although no specific antidote exists for inhalation-induced lung injury, supportive treatments have advanced significantly (41). Recent innovations, such as high-volume hemofiltration and extracorporeal membrane oxygenation, have shown promising results (28, 42). Studies also suggest that nebulized sodium bicarbonate may aid in treating inhalation lung injury by neutralizing acid production in the pulmonary epithelium (43). Current treatment strategies mainly focus on supplemental oxygen, protective ventilation, nebulized bronchodilators, and intravenous corticosteroids (28, 44).

Acute respiratory distress syndrome is a severe clinical syndrome characterized by progressive respiratory difficulty and persistent hypoxemia (45). It can arise from various causes, including inhalation of toxic gases, pneumonia, bacterial, viral, or fungal infections, sepsis, severe trauma, and burns (46, 47). The disease involves injury to both the alveolar epithelium and capillary endothelium, resulting in inflammatory exudation from the alveolar capillaries, activation of macrophages and neutrophils, and the development of pulmonary edema and atelectasis (48). The clinical manifestations of ARDS vary depending on the underlying cause, exhibiting distinct physiological features that influence the patient’s response to treatment (49). Systemic corticosteroids have been considered a potential therapy to control inflammation and improve survival in ARDS (33, 50). Recent clinical guidelines recommend corticosteroid use for ARDS management, based on moderate evidence. However, no multicenter randomized controlled trials have evaluated glucocorticoid treatment specifically for ARDS caused by chlorine gas (36). While various treatment regimens for ARDS from other causes have been tested, the results remain controversial. Meduri et al. demonstrated that a low-dose, prolonged methylprednisolone regimen alleviated systemic inflammation in patients with severe early ARDS (within 72 h of symptom onset), improving lung and extrapulmonary organ function, and reducing the duration of mechanical ventilation and ICU stay. In contrast, methylprednisolone administration between 7 and 14 days after ARDS diagnosis was associated with significantly increased 60- and 180-day mortality rate (51). Several studies have employed a high-dose protocol (1,000 mg/day for the first 3 days) followed by a moderate taper (2 mg/kg/day over 2 months) (52). Zeiner et al. found that high-dose methylprednisolone improved respiratory function and survival in 65% of patients with refractory COVID-19-related ARDS (53). However, other studies indicate that initial high-dose methylprednisolone therapy followed by tapering may have adverse effects on patients with ARDS (54). Therefore, corticosteroid treatment should be individualized, based on the underlying pathogenesis and severity of ARDS.

In this study, the patient exhibited acute symptoms commonly reported in other cases, including chest tightness, tachycardia, shortness of breath, and the need for oxygen, all indicative of chlorine inhalation-related complications such as chemical pneumonitis, pulmonary edema, and ARDS. The management of chlorine gas inhalation injury is predominantly conservative. Currently, no universally accepted guidelines exist regarding the dosage, duration, or course of glucocorticoid therapy for patients with ARDS (55). The available data on systemic steroid use are limited to a small number of case reports. Table 2 summarizes various case reports involving chlorine exposure, steroid use, and subsequent outcomes. One case, reported by Bleifuss et al., was managed with standard ARDS care, including inhaled epoprostenol, bronchodilators, intravenous dexamethasone, and prone positioning (56). Chian et al. described a case with a prolonged 176-day hospital stay due to ARDS, where the patient’s condition improved with high-dose corticosteroid treatment, a tapering regimen, and extracorporeal life support during ICU care, followed by rehabilitation (57). Similarly, Babu et al. reported a case with a 28-day hospital stay and a favorable prognosis, managed with a 2 mg/kg methylprednisolone bolus on day 10 of intubation, followed by 2 mg/kg in divided doses (44). Conversely, Petilla et al. reported a case initially treated with intravenous ceftriaxone and methylprednisolone (250 mg/day) without adjunctive oxygen or antidote treatment, which resulted in prolonged ICU stay and recurrent bilateral pneumothorax, requiring bilateral excision of emphysematous bullae and pleurodesis (58). In our patient, high-dose methylprednisolone with a tapering regimen and non-invasive ventilatory support were employed. Due to a secondary Candida albicans infection, the patient received the fluconazole for antifungal treatment, cefoperazone sulbactam for antibacterial treatment, and continued with corticosteroid therapy. With active treatment, the patient’s prognosis remained relatively favorable.

**TABLE 2:** Published cases in chloride inhalational injury.

Case report	Age	Sex	Exposure	Onset	Management	Outcome	Systemic steroid	Follow-up
Bleifuss et al. (56)	Male	75	Mixture of calcium hypochlorite powder with water	2 h after exposure	Medically managed and underwent prone positioning	Discharged 22 days later	Intravenous dexamethasone, while the dosage and duration were not reported.	Cardiopulmonary Exercise Testing (CPET) 1 month later showed normal breathing exercise tolerance.
Pham et al. (75)	Male	43	Mixture of TCCA 90% (Trichloroisocyanuric acid) and Aquafit chlorine powder 70% (Calcium hypochlorite)	3 h after exposure	Medically managed	Discharged 7 days later	Intravenous methylprednisolone (80 mg every 24 h) for 6 days.	NA
Mustafa et al. (76)	Male	65	Inhalation of aerosols contaminated with *Mycobacterium avium* complex (MAC) from poorly maintained hot tubs.	Worsening after 6 months	Medically managed	NA	Starting at 40 mg prednisolone orally for 7 days and then decreasing by 5 mg weekly until completion.	Follow-up pulmonary function tests in 2 months and a repeat CT scan in 3 months showed normal results.
Wang et al. (77)	Female	28	Mix and dissolve the disinfectant tablets containing dichloroisocyanuric acid and trichloroisocyanuric acid in water.	10 h after exposure	Awake self-prone positioning (ASPP) combined with high-flow nasal oxygen therapy.	On the 6th day, the patient was transferred to the general ward for further treatment.	Intravenous dexamethasone, while the dosage and duration were not reported.	NA
Baig and Salahuddin (25)	Male	38	Chlorine gas leakage	During the course of Emergency Department stay	Bilateral tube thoracostomy	After 10 days of MICU stay	NA	NA
Akhlaq et al. (78)	Male	NA (young)	Leakage of disinfectant or insecticide	Worsening after 5 months	ICU stay, and bilateral tube thoracostomies	Discharged 10 days later	NA	NA
Katano et al. (79)	Female	43	Mixture of household bleach and vinegar.	1 day after admission	Medically managed	Discharged 6 days later	NA	NA
Harischandra et al. (80)	Female	11	Chlorine gas during the disinfection process at a swimming pool.	Upon admission	ECMO therapy	Discharged 6 days later	Intravenous methylprednisolone, but the dosage and duration were not reported.	Chest radio graphs and lung function tests 6 months later showed normal results.
Cromie and Flannigan, (27)	Male	9	Mixture of sodium hypochlorite and red diesel releases gas at the campfire site	Worsened after over 24 h	Medically managed	Discharged home 5 days after presentation	A 5-day course of steroids, while the dosage and duration were not reported.	After 6 weeks of follow-up at the outpatient clinic with very good condition.
Li et al. (12)	Female	13	TCCA powder was accidentally dropped into a community pool	Worsening after 2 h	Medically managed; Continuous drainage tube was placed	Symptoms gradually improved after 18 days.	Glucocorticoid support treatment, while the dosage and duration were not reported.	NA
	Male	15		Worsening 3 h after admission	No special treatment	Discharged 15 days later	NA	NA
Chian et al. (57)	Male	23	Exposure to the smoke generated by a smoke grenade without any protection	ARDS occurs 48 h after exposure	Extracorporeal life support (ECLS) and bilateral pulmonary consolidations	Discharged after 176 days	High-dose corticosteroid treatment, intravenous methylprednisolone 500 mg/d with a tapering regime.	NA
Babu et al. (44)	Female	23	Chlorine fumes at a community swimming pool	Immediate	ICU stay, and tracheostomy	Discharged 28 days later	2 mg/kg methylprednisolone bolus on day 10 of intubation, followed by 2 mg/kg in two divided doses per day.	NA
Kanne et al. (81)	Male	23	Leakage of chlorine gas in the maintenance room	36 h after exposure	Medically managed	Full recovery after over 5 months.	Oral glucocorticoids, but the dosage and duration were unkonown.	NA
Vohra and Clark (82)	Female	9	Mix and dissolve the chemical particles of the purification tablets in a pool.	Worsening after 2 days	Medically managed	Discharged from the hospital on the second day	No steroids, inhaled sodium bicarbonate (NaHCO_3_), or antibiotics.	The telephone follow-up after 4 months indicated that there was no remaining respiratory discomfort.
Akdur et al. (26)	Male	26	Gas produced by inhaling the mixture of household cleaning products	2 days after exposure	Medically managed	Discharged 6 days later	Intravenous dexamethasone, while the dosage and duration were not reported	Great PFTs and β agonist response after 6 months.
Pettilä et al. (58)	Male	18	Zinc chloride inhalation from smoke grenade	ARDS developed on day 2	ICU stay, and bilateral excision of emphysematic bullae and pleurodesis.	NA	Intravenous methylprednisolone 250 mg/day.	NA

According to the latest Berlin criteria, the diagnosis of ARDS requires a comprehensive clinical evaluation, including the acute onset of respiratory distress within 1 week, bilateral patchy infiltrates on chest X-ray and CT scans, and a PaO_2_/FiO_2_ ≤ 300 mmHg or SpO_2_/FiO_2_ ≤ 315 (45). The SpO_2_/FiO_2_ ratio can serve as an alternative indicator of hypoxemia when arterial blood gas sampling is not feasible (59, 60). Studies have confirmed that the SpO_2_/FiO_2_ ratio closely correlates with PaO_2_/FiO_2_, aiding in earlier ARDS diagnosis and enabling timely implementation of lung-protective ventilation and fluid management strategies (61, 62). Since the patient could not tolerate arterial blood gas sampling, blood gas analysis was performed during two emergency treatment sessions. Dosage adjustments were made based on a comprehensive assessment of the patient’s clinical symptoms, SpO_2_/FiO_2_ ratio, CT imaging, and laboratory results (50, 59). After receiving low-dose steroids, the patient developed a Candida infection and was treated with fluconazole for antifungal therapy and cefoperazone sulbactam for antibacterial treatment. In patients receiving reduced glucocorticoid dosages, caution is necessary to prevent secondary infections, and the temporary use of antibiotics and antifungal agents may be required.

Glucocorticoids exert potent anti-inflammatory and immunosuppressive effects by binding to intracellular glucocorticoid receptors (GR), playing a pivotal role in modulating immune responses (63–66). These effects include a strong inhibition of neutrophil and macrophage chemotaxis, phagocytosis, and bactericidal activity, achieved through decreased expression of ROS, cyclooxygenase-2 (COX-2), and inducible nitric oxide synthase (iNOS) (67, 68). The glucocorticoid-GR complex directly prevents the activation of toll-like receptor-2 (TLR-2), NF-κB, and activator protein-1, thereby inhibiting the expression of downstream proinflammatory genes (69, 70). Additionally, glucocorticoids suppress the expression of co-stimulatory molecules, cytokines, and chemokines in T cells, leading to potent T cell inhibition (71). Furthermore, by reducing IL-12 and IFN-γ production in macrophages and dendritic cells, glucocorticoids diminish Th1 cell activation while promoting Th17 differentiation. This shift enhances the expression of the anti-inflammatory mediator TGF-β and triggers a transition from Th1 to Th2 immunity [(65), (71, 72)]. Since fungal infections primarily rely on cellular immunity for prevention, the suppression of this immunity by glucocorticoids heightens the risk of latent infections (35, 73). As glucocorticoid dosage is tapered, the immune system gradually recovers, but this process is slow and insufficient to effectively combat fungal antigens accumulated during the period of immunosuppression, leading to the reactivation of latent infections (73, 74).

This case provides valuable insights into the rationale for glucocorticoid dosage adjustments and underscores the potential benefit of early systemic corticosteroid use in patients with chlorine-induced lung injury. However, as a single case report, the findings cannot be generalized, and further investigation through large-scale, multicenter studies is necessary to determine the effectiveness of systemic corticosteroids for chlorine-induced lung injury.

## 4 Conclusion

The early application of a high-dose corticosteroid tapering regimen combined with non-invasive mechanical ventilation can effectively manage ARDS caused by chlorine gas inhalation. When arterial blood gas sampling is not feasible, the corticosteroid dosage adjustments are based on a comprehensive assessment of the clinical symptoms, SpO_2_/FiO_2_ ratio, CT imaging, and laboratory results. Additionally, vigilance for secondary infections during treatment is crucial, requiring prompt intervention for symptoms such as fever, purulent sputum, or new and progressing lung abnormalities on imaging. More importantly, large-scale randomized controlled studies are needed to explore the application of systemic corticosteroids in chlorine-induced lung injury.

## Data Availability

The original contributions presented in the study are included in the article/supplementary material, further inquiries can be directed to the corresponding author.

## References

[1] He H Li F Liu K Zhan J Wang X Lai C The disinfectant residues promote the leaching of water contaminants from plastic pipe particles. *Environ Pollut*. (2023). 327:121577. 10.1016/j.envpol.2023.121577 37023886

[2] Oliveira IM Gomes IB Simões LC Simões M. Chlorinated cyanurates and potassium salt of peroxymonosulphate as antimicrobial and antibiofilm agents for drinking water disinfection. *Sci Total Environ*. (2022) 811:152355. 10.1016/j.scitotenv.2021.152355 34921876

[3] Yang Y Xu Z Xiao Z Luo J Wu Y Lin Z Research on the mechanism of flumequine degradation by ultraviolet light activated trichloroisocyanuric acid. *Environ Res*. (2025) 281:121976. 10.1016/j.envres.2025.121976 40436195

[4] Peng F Wang Y Lu Y Yang Z Li H. Formation and control of disinfection by-products during the trichloroisocyanuric acid disinfection in swimming pool water. *Environ Pollut*. (2024) 346:123536. 10.1016/j.envpol.2024.123536 38365079

[5] Yang L Schmalz C Zhou J Zwiener C Chang VW Ge L An insight of disinfection by-product (DBP) formation by alternative disinfectants for swimming pool disinfection under tropical conditions. *Water Res*. (2016) 101:535–46. 10.1016/j.watres.2016.05.088 27300590

[6] Chhabra G Ahmad N. Trichloroisocyanuric acid, a swimming pool disinfectant: new developments and role in UV-induced skin inflammation†. *Photochem Photobiol*. (2023) 99:869–71. 10.1111/php.13700 36004539

[7] Snell JA Vaishampayan P Dickinson SE Jandova J Wondrak GT. The drinking water and swimming pool disinfectant trichloroisocyanuric acid causes chlorination stress enhancing solar UV-induced inflammatory gene expression in AP-1 transgenic SKH-1 luciferase reporter mouse skin. *Photochem Photobiol*. (2023) 99:835–43. 10.1111/php.13675 35841216 PMC10321141

[8] Hoyle GW Svendsen ER. Persistent effects of chlorine inhalation on respiratory health. *Ann N Y Acad Sci*. (2016) 1378:33–40. 10.1111/nyas.13139 27385061 PMC5063681

[9] Achanta S Gentile MA Albert CJ Schulte KA Pantazides BG Crow BS Recapitulation of human pathophysiology and identification of forensic biomarkers in a translational model of chlorine inhalation injury. *Am J Physiol Lung Cell Mol Physiol*. (2024) 326:L482–95. 10.1152/ajplung.00162.2023 38318664 PMC11281795

[10] de Genaro IS de Almeida FM Dos Santos Lopes FDTQ Kunzler DCH Tripode BGB Kurdejak A Low-dose chlorine exposure impairs lung function, inflammation and oxidative stress in mice. *Life Sci*. (2021) 267:118912. 10.1016/j.lfs.2020.118912 33338503

[11] Achanta S Jordt SE. Toxic effects of chlorine gas and potential treatments: a literature review. *Toxicol Mech Methods*. (2021) 31:244–56. 10.1080/15376516.2019.1669244 31532270 PMC7108975

[12] Li B Jia L Shao D Liu H Nie S Tang W Pneumomediastinum from acute inhalation of chlorine gas in 2 young patients. *Am J Emerg Med.* (2011) 29:357.e1-4. 10.1016/j.ajem.2010.04.007 20627215

[13] Jonasson S Koch B Bucht A. Inhalation of chlorine causes long-standing lung inflammation and airway hyperresponsiveness in a murine model of chemical-induced lung injury. *Toxicology*. (2013) 303:34–42. 10.1016/j.tox.2012.10.022 23146759

[14] Malaviya R Gardner CR Rancourt RC Smith LC Abramova EV Vayas KN Lung injury and oxidative stress induced by inhaled chlorine in mice is associated with proinflammatory activation of macrophages and altered bioenergetics. *Toxicol Appl Pharmacol*. (2023) 461:116388. 10.1016/j.taap.2023.116388 36690086 PMC9960611

[15] Martin JG Campbell HR Iijima H Gautrin D Malo JL Eidelman DH Chlorine-induced injury to the airways in mice. *Am J Respir Crit Care Med*. (2003) 168:568–74. 10.1164/rccm.200201-021OC 12724121

[16] Dubey S Yu Z Stephens EM Lazrak A Ahmad I Aggarwal S Oxidative damage to lung mitochondrial DNA is a key contributor to the development of chemical lung injury. *Redox Biol*. (2025) 82:103624. 10.1016/j.redox.2025.103624 40209617 PMC12013491

[17] Honavar J Samal AA Bradley KM Brandon A Balanay J Squadrito GL Chlorine gas exposure causes systemic endothelial dysfunction by inhibiting endothelial nitric oxide synthase-dependent signaling. *Am J Respir Cell Mol Biol*. (2011) 45:419–25. 10.1165/rcmb.2010-0151OC 21131444 PMC3175567

[18] Lee J Giordano S Zhang J. Autophagy, mitochondria and oxidative stress: cross-talk and redox signalling. *Biochem J*. (2012) 441:523–40. 10.1042/BJ20111451 22187934 PMC3258656

[19] Murashevych B Maslak H Girenko D Abraimova O Netronina O Shvets V. The effect of hypochlorous acid inhalation on the activity of antioxidant system enzymes in rats of different ages. *Free Radic Res*. (2024) 58:441–57. 10.1080/10715762.2024.2386688 39073910

[20] Woods CG Fu J Xue P Hou Y Pluta LJ Yang L Dose-dependent transitions in Nrf2-mediated adaptive response and related stress responses to hypochlorous acid in mouse macrophages. *Toxicol Appl Pharmacol*. (2009) 238:27–36. 10.1016/j.taap.2009.04.007 19376150 PMC2697450

[21] Wigenstam E Elfsmark L Koch B Bucht A Jonasson S. Acute respiratory changes and pulmonary inflammation involving a pathway of TGF-β1 induction in a rat model of chlorine-induced lung injury. *Toxicol Appl Pharmacol*. (2016) 309:44–54. 10.1016/j.taap.2016.08.027 27586366

[22] Zhao CQ Liu JZ Liu MM Ren XT Kong DQ Peng J Heterogeneity of T cells and macrophages in chlorine-induced acute lung injury in mice using single-cell RNA sequencing. *Inhal Toxicol*. (2022) 34:399–411. 10.1080/08958378.2022.2134526 36260290

[23] Bos LDJ Ware LB. Acute respiratory distress syndrome: causes, pathophysiology, and phenotypes. *Lancet*. (2022) 400:1145–56. 10.1016/S0140-6736(22)01485-4 36070787

[24] Zellner T Eyer F. Choking agents and chlorine gas - History, pathophysiology, clinical effects and treatment. *Toxicol Lett*. (2020) 320:73–9. 10.1016/j.toxlet.2019.12.005 31811912

[25] Baig MA Salahuddin M. Pneumomediastinum in an accidental chlorine gas exposed victim. *J Coll Physicians Surg Pak*. (2022) 32:S73–5. 10.29271/jcpsp.2022.Supp1.S73 35633019

[26] Akdur O Durukan P Ikizceli I Ozkan S Avsarogullari L. A rare complication of chlorine gas inhalation: pneumomediastinum. *Emerg Med J*. (2006) 23:e59. 10.1136/emj.2006.040022 17057124 PMC2464375

[27] Cromie S Flannigan C. Chemical pneumonitis in a 9-year-old following chlorine gas exposure. *BMJ Case Rep*. (2019) 12:e229281. 10.1136/bcr-2019-229281 31366613 PMC6677986

[28] Wang L Wu D Wang J. Chlorine gas inhalation manifesting with severe acute respiratory distress syndrome successfully treated by high-volume hemofiltration: a case report. *Medicine*. (2018) 97:e11708. 10.1097/MD.0000000000011708 30045333 PMC6078753

[29] Fujishima S. Current corticosteroid therapeutic strategy for community-acquired pneumonia in adults: indications, dosage, and timing. *J Intensive Care*. (2025) 13:37. 10.1186/s40560-025-00809-8 40598558 PMC12210833

[30] Zhang J Ge P Liu J Luo Y Guo H Zhang G Glucocorticoid treatment in acute respiratory distress syndrome: an overview on mechanistic insights and clinical benefit. *Int J Mol Sci*. (2023) 24:12138. 10.3390/ijms241512138 37569514 PMC10418884

[31] Zhao Y Yao Z Xu S Yao L Yu Z. Glucocorticoid therapy for acute respiratory distress syndrome: current concepts. *J Intensive Med*. (2024) 4:417–32. 10.1016/j.jointm.2024.02.002 39310055 PMC11411438

[32] Leung PB Davis AM Davis J. Corticosteroids for sepsis, acute respiratory distress syndrome, or community-acquired pneumonia. *JAMA*. (2025) 333:421–2. 10.1001/jama.2024.24537 39774599

[33] Meduri GU Bridges L Shih MC Marik PE Siemieniuk RAC Kocak M. Prolonged glucocorticoid treatment is associated with improved ARDS outcomes: analysis of individual patients’ data from four randomized trials and trial-level meta-analysis of the updated literature. *Intensive Care Med*. (2016) 42:829–40. 10.1007/s00134-015-4095-4 26508525

[34] Pillay J Flikweert AW van Meurs M Grootenboers MJ van der Sar-van der Brugge S van der Voort PHJ Extracellular matrix turnover in severe COVID-19 is reduced by corticosteroids. *Respir Res*. (2025) 26:32. 10.1186/s12931-025-03098-9 39844140 PMC11755962

[35] Pirracchio R Venkatesh B Legrand M. Low-dose corticosteroids for critically ill adults with severe pulmonary infections: a review. *JAMA*. (2024) 332:318–28. 10.1001/jama.2024.6096 38865154 PMC13475641

[36] Qadir N Sahetya S Munshi L Summers C Abrams D Beitler J An update on management of adult patients with acute respiratory distress syndrome: an official american thoracic society clinical practice guideline. *Am J Respir Crit Care Med*. (2024) 209:24–36. 10.1164/rccm.202311-2011ST 38032683 PMC10870893

[37] Li G Chen D Gao F Huang W Wang J Li Y Efficacy of corticosteroids in patients with acute respiratory distress syndrome: a meta-analysis. *Ann Med*. (2024) 56:2381086. 10.1080/07853890.2024.2381086 39165240 PMC11340212

[38] Kiser TH Sevransky JE Krishnan JA Tonascia J Wise RA Checkley W A survey of corticosteroid dosing for exacerbations of chronic obstructive pulmonary disease requiring assisted ventilation. *Chronic Obstr Pulm Dis*. (2017) 4:186–93. 10.15326/jcopdf.4.3.2016.0168 28848930 PMC5556910

[39] Atalla A Shulman J Rose J Lynch M. Trends in chlorine and chloramine gas exposures reported to United States poison centers. *Clin Toxicol*. (2024) 62:589–95. 10.1080/15563650.2024.2390139 39263698 PMC12184804

[40] White CW Martin JG. Chlorine gas inhalation: human clinical evidence of toxicity and experience in animal models. *Proc Am Thorac Soc*. (2010) 7:257–63. 10.1513/pats.201001-008SM 20601629 PMC3136961

[41] Summerhill EM Hoyle GW Jordt SE Jugg BJ Martin JG Matalon S An Official American thoracic society workshop report: chemical inhalational disasters. biology of lung injury, development of novel therapeutics, and medical preparedness. *Ann Am Thorac Soc*. (2017) 14:1060–72. 10.1513/AnnalsATS.201704-297WS 28418689 PMC5529138

[42] Vajner JE Lung D. Case files of the University of California San Francisco Medical Toxicology Fellowship: acute chlorine gas inhalation and the utility of nebulized sodium bicarbonate. *J Med Toxicol*. (2013) 9:259–65. 10.1007/s13181-013-0309-8 23719961 PMC3770993

[43] Aslan S Kandiş H Akgun M Cakir Z Inandi T Görgüner M. The effect of nebulized NaHCO3 treatment on “RADS” due to chlorine gas inhalation. *Inhal Toxicol*. (2006) 18:895–900. 10.1080/08958370600822615 16864407

[44] Babu RV Cardenas V Sharma G. Acute respiratory distress syndrome from chlorine inhalation during a swimming pool accident: a case report and review of the literature. *J Intensive Care Med*. (2008) 23:275–80. 10.1177/0885066608318471 18508837

[45] Matthay MA Arabi Y Arroliga AC Bernard G Bersten AD Brochard LJ A new global definition of acute respiratory distress syndrome. *Am J Respir Crit Care Med*. (2024) 209:37–47. 10.1164/rccm.202303-0558WS 37487152 PMC10870872

[46] Grasselli G Calfee CS Camporota L Poole D Amato MBP Antonelli M ESICM guidelines on acute respiratory distress syndrome: definition, phenotyping and respiratory support strategies. *Intensive Care Med*. (2023) 49:727–59. 10.1007/s00134-023-07050-7 37326646 PMC10354163

[47] Norisue Y Yamamoto R Yamakawa H Hibino M Nagai T Fujimoto Y Incidence of diffuse parenchymal lung disease in patients meeting the Berlin definition of acute respiratory distress syndrome on mechanical ventilation. *ERJ Open Res*. (2025) 11:01296–2024. 10.1183/23120541.01296-2024 40959165 PMC12434489

[48] Choi WI Shehu E Lim SY Koh SO Jeon K Na S Markers of poor outcome in patients with acute hypoxemic respiratory failure. *J Crit Care*. (2014) 29:797–802. 10.1016/j.jcrc.2014.05.017 24997724

[49] Ma W Tang S Yao P Zhou T Niu Q Liu P Advances in acute respiratory distress syndrome: focusing on heterogeneity, pathophysiology, and therapeutic strategies. *Signal Transduct Target Ther*. (2025) 10:75. 10.1038/s41392-025-02127-9 40050633 PMC11885678

[50] Seam N Meduri GU Wang H Nylen ES Sun J Schultz MJ Effects of methylprednisolone infusion on markers of inflammation, coagulation, and angiogenesis in early acute respiratory distress syndrome. *Crit Care Med*. (2012) 40:495–501. 10.1097/CCM.0b013e318232da5e 21983371 PMC10802149

[51] Meduri GU Golden E Freire AX Taylor E Zaman M Carson SJ Methylprednisolone infusion in early severe ARDS: results of a randomized controlled trial. *Chest*. (2007) 131:954–63. 10.1378/chest.06-2100 17426195

[52] Steinberg KP Hudson LD Goodman RB Hough CL Lanken PN Hyzy R Efficacy and safety of corticosteroids for persistent acute respiratory distress syndrome. *N Engl J Med*. (2006) 354:1671–84. 10.1056/NEJMoa051693 16625008

[53] Zeiner C Schröder M Metzner S Herrmann J Notz Q Hottenrott S High-dose methylprednisolone pulse therapy during refractory COVID-19 acute respiratory distress syndrome: a retrospective observational study. *BMC Pulm Med*. (2023) 23:368. 10.1186/s12890-023-02664-5 37789367 PMC10546709

[54] Takaki M Ichikado K Kawamura K Gushima Y Suga M. The negative effect of initial high-dose methylprednisolone and tapering regimen for acute respiratory distress syndrome: a retrospective propensity matched cohort study. *Crit Care*. (2017) 21:135. 10.1186/s13054-017-1723-0 28592332 PMC5463340

[55] Yang JW Jiang P Wang WW Wen ZM Mao B Lu HW The controversy about the effects of different doses of corticosteroid treatment on clinical outcomes for acute respiratory distress syndrome patients: an observational Study. *Front Pharmacol*. (2021) 12:722537. 10.3389/fphar.2021.722537 34393800 PMC8358143

[56] Bleifuss W Pepin L Lock S Kaluzniak H Kempainen R. Chlorine gas induced acute respiratory distress syndrome due to pool shock. *Am J Emerg Med.* (2025) 93:236.e3–236.e5. 10.1016/j.ajem.2025.04.001 40187988

[57] Chian CF Wu CP Chen CW Su WL Yeh CB Perng WC. Acute respiratory distress syndrome after zinc chloride inhalation: survival after extracorporeal life support and corticosteroid treatment. *Am J Crit Care*. (2010) 19:86–90. 10.4037/ajcc2009908 19304566

[58] Pettilä V Takkunen O Tukiainen P. Zinc chloride smoke inhalation: a rare cause of severe acute respiratory distress syndrome. *Intensive Care Med*. (2000) 26:215–7. 10.1007/s001340050049 10784312

[59] Catoire P Tellier E de la Rivière C Beauvieux MC Valdenaire G Galinski M Assessment of the SpO2/FiO2 ratio as a tool for hypoxemia screening in the emergency department. *Am J Emerg Med*. (2021) 44:116–20. 10.1016/j.ajem.2021.01.092 33588251 PMC7865090

[60] Rice TW Wheeler AP Bernard GR Hayden DL Schoenfeld DA Ware LB Comparison of the SpO2/FIO2 ratio and the PaO2/FIO2 ratio in patients with acute lung injury or ARDS. *Chest*. (2007) 132:410–7. 10.1378/chest.07-0617 17573487

[61] Kim JH Baek AR Lee SI Kim WY Na YS Lee BY ROX index and SpO2/FiO2 ratio for predicting high-flow nasal cannula failure in hypoxemic COVID-19 patients: a multicenter retrospective study. *PLoS One*. (2022) 17:e0268431. 10.1371/journal.pone.0268431 35551328 PMC9098056

[62] Satici MO Islam MM Satici C Uygun CN Ademoglu E Altunok I The role of a noninvasive index ‘Spo2/ Fio2’ in predicting mortality among patients with COVID-19 pneumonia. *Am J Emerg Med*. (2022) 57:54–9. 10.1016/j.ajem.2022.04.036 35525158 PMC9044731

[63] Li CC Munitic I Mittelstadt PR Castro E Ashwell JD. Suppression of dendritic cell-derived IL-12 by endogenous glucocorticoids is protective in LPS-induced sepsis. *PLoS Biol*. (2015) 13:e1002269. 10.1371/journal.pbio.1002269 26440998 PMC4595142

[64] Oh KS Patel H Gottschalk RA Lee WS Baek S Fraser IDC Anti-inflammatory chromatinscape suggests alternative mechanisms of glucocorticoid receptor action. *Immunity.* (2017) 47:298–309.e5. 10.1016/j.immuni.2017.07.012 28801231 PMC5572836

[65] Taves MD Ashwell JD. Glucocorticoids in T cell development, differentiation and function. *Nat Rev Immunol*. (2021) 21:233–43. 10.1038/s41577-020-00464-0 33149283

[66] Cannarile L Zollo O D’Adamio F Ayroldi E Marchetti C Tabilio A Cloning, chromosomal assignment and tissue distribution of human GILZ, a glucocorticoid hormone-induced gene. *Cell Death Differ*. (2001) 8:201–3. 10.1038/sj.cdd.4400798 11313722

[67] Amratia DA Viola H Ioachimescu OC. Glucocorticoid therapy in respiratory illness: bench to bedside. *J Investig Med*. (2022) 70:1662–80. 10.1136/jim-2021-002161 35764344 PMC9726965

[68] Masferrer JL Seibert K Zweifel B Needleman P. Endogenous glucocorticoids regulate an inducible cyclooxygenase enzyme. *Proc Natl Acad Sci U S A*. (1992) 89:3917–21. 10.1073/pnas.89.9.3917 1570314 PMC525602

[69] Busillo JM Azzam KM Cidlowski JA. Glucocorticoids sensitize the innate immune system through regulation of the NLRP3 inflammasome. *J Biol Chem*. (2011) 286:38703–13. 10.1074/jbc.M111.275370 21940629 PMC3207479

[70] Ingawale DK Mandlik SK. New insights into the novel anti-inflammatory mode of action of glucocorticoids. *Immunopharmacol Immunotoxicol*. (2020) 42:59–73. 10.1080/08923973.2020.1728765 32070175

[71] Ronchetti S Migliorati G Bruscoli S Riccardi C. Defining the role of glucocorticoids in inflammation. *Clin Sci*. (2018) 132:1529–43. 10.1042/CS20171505 30065045

[72] Banuelos J Lu NZ. A gradient of glucocorticoid sensitivity among helper T cell cytokines. *Cytokine Growth Factor Rev*. (2016) 31:27–35. 10.1016/j.cytogfr.2016.05.002 27235091 PMC5050075

[73] Busillo JM Cidlowski JA. The five Rs of glucocorticoid action during inflammation: ready, reinforce, repress, resolve, and restore. *Trends Endocrinol Metab*. (2013) 24:109–19. 10.1016/j.tem.2012.11.005 23312823 PMC3667973

[74] Mellinghoff SC Thelen M Bruns C Garcia-Marquez M Hartmann P Lammertz T T-cells of invasive candidiasis patients show patterns of T-cell-exhaustion suggesting checkpoint blockade as treatment option. *J Infect*. (2022) 84:237–47. 10.1016/j.jinf.2021.12.009 34921845

[75] Pham DT Dao BN Nguyen DT Van Dang B Le DT Do HM A rare case of acute respiratory distress syndrome due to chlorine gas inhalation: rapid progression with favorable ICU outcome. *Respir Med Case Rep*. (2025) 53:102148. 10.1016/j.rmcr.2024.102148 39760045 PMC11700285

[76] Mustafa R Gadallah M Elfaki A Asuquo B. Chlorine-induced lung injury from hot tub exposure. *Cureus*. (2024) 16:e70025. 10.7759/cureus.70025 39449885 PMC11499000

[77] Wang F Liu F Lu H. Successful treatment of 1 patient with chlorine-induced ARDS using awake self-prone positioning and nasal high-flow oxygen: a case report. *Medicine*. (2024) 103:e36995. 10.1097/MD.0000000000036995 38241571 PMC10798783

[78] Akhlaq S Ejaz T Aziz A Ahmed A. Spontaneous pneumomediastinum in accidental chlorine gas inhalational injury: case report and review of literature. *BMJ Case Rep*. (2021) 14:e236549. 10.1136/bcr-2020-236549 34330735 PMC8327745

[79] Katano T Murao H Kato T Kubo A Ito S. A case of acute inhalation injury caused by premeditated chlorine gas exposure. *Respirol Case Rep*. (2021) 9:e00743. 10.1002/rcr2.743 33791101 PMC7996109

[80] Harischandra T Withanaarachchi K Piyasiri B Wickramasuriya H Piyasiri G Firmin R. Successful use of extracorporeal membrane oxygenation in acute respiratory distress syndrome following accidental chlorine gas inhalation at a swimming pool. *Perfusion*. (2020) 35:543–5. 10.1177/0267659120922013 32441230

[81] Kanne JP Thoongsuwan N Parimon T Stern EJ. Trauma cases from Harborview Medical Center. Airway injury after acute chlorine exposure. *AJR Am. J. Roentgenol.* (2006) 186:232–3. 10.2214/ajr.05.0202 16357407

[82] Vohra R Clark RF. Chlorine-related inhalation injury from a swimming pool disinfectant in a 9-year-old girl. *Pediatr Emerg Care*. (2006) 22:254–7. 10.1097/01.pec.0000210173.56010.c1 16651917

